# Past and future regret and missed opportunities: an experimental approach on separate evaluation and different time frames

**DOI:** 10.1186/s41155-017-0074-8

**Published:** 2017-09-21

**Authors:** Luisa Papé, Luis F. Martinez

**Affiliations:** Nova School of Business and Economics, Universidade Nova de Lisboa, Campus de Campolide, 1099-032 Lisboa, Portugal

**Keywords:** Regret, Decision-making, Past opportunity, Future opportunity, Emotion

## Abstract

Decisions often imply trade-offs that force people to accept missing an opportunity in the past or in the future. However, it is not fully clear whether a past miss or a future miss elicits more regret. In a direct comparison, previous research had found support for the greater impact of future misses. In an experimental study with 216 participants, we replicated and extended previous research by testing the strength of the future miss in a separate evaluation and with different periods. Results show that, when evaluated separately, future misses caused less regret than past misses. However, future misses made participants change their feelings of regret more intensely than past misses did. Also, regret levels did not decrease when future misses were further away. Our findings support the strength of future misses on regret but also show contrasting effects when evaluated separately.

## Background

*“All negativity is caused by an accumulation of psychological time and denial of the present. Unease, anxiety, tension, stress worry – all forms of fear – are caused by too much future and not enough presence. Guilt, regret, resentment, grievances, sadness, bitterness, and all forms of nonforgiveness are caused by too much past, and not enough presence* *.”* (Tolle, 2012, p. 127)

According to Tolle (2012), spiritual teacher and author, the constant need of the mind to remember the past and to think about the future is the main factor that prevents us from experiencing inner peace and personal happiness. It is in fact a very special ability that is still assumed to be a unique ability in which only humans can excel (Cheke & Clayton, 2010; Roberts, 2002). Even though it is responsible for emotional ups and downs, such as joy or regret, and prevents us from experiencing this inner peace, it still has a positive aspect as it drives us to constantly improve and learn from our successes and mistakes (Epstude & Roese, 2008; Roese, 1997). This, in turn, increases the quality of future decisions and enables overall improved living conditions (Zeelenberg, 1999).

The decisions that everyone has to make—such as those involving career, private life, or even leisure time—could involve strong emotional experiences. These includes feelings about the respective decision, such as fear, anxiety about the consequences, and regarding the expectations about feelings that may occur after the outcome, such as relief or sadness. Of all the emotions a person can experience, regret has received the most attention in past research (Connolly & Zeelenberg, 2002). Zeelenberg and Pieters (2007) showed this by collecting academic publications on the topic of regret from 1945 to 2005, and they concluded that from the 1990s, publications on this topic increased dramatically. This clearly shows an increased interest in regret over the last decades.

One reason for the increased interest is that regret has a significant influence on decision-making. It is therefore one of the most frequently experienced emotions in human emotional life and especially occurs when individuals hold themselves personally responsible for the undesirable outcome (Connolly & Zeelenberg, 2002; Zeelenberg, 1999).

This study aims to shed further light on the findings of previous research by Shani, Danziger, and Zeelenberg (2015). As a first step, an overview of important theoretical concepts for the topic is presented. In an experimental study, we seek to extend the findings of Shani et al. (2015) by modifying some of their experiments in order to add new insights and variables to the current literature in this field of research. The overall question is whether missed opportunities in the future elicit more regret than missed opportunities in the past.

### Regret

Regret can be defined as an emotion that occurs when a person is thinking about how a current situation would have been better if a different decision had been made. This negative emotion emerges from an undesirable evaluation process of a certain decision. Accordingly, the feeling leads to self-blaming for making the wrong decision and a desire to reverse the prevailing situation (Zeelenberg & Pieters, 2007). Furthermore, it is emphasized that this specific emotion is generally not categorized as one of the basic emotions such as happiness, anger, or sadness, which even a newborn can experience. It is rather a comparison-based emotion (van Dijk & Zeelenberg, 2005), a more complex pattern where the capacity to formulate another set of circumstances is necessary. These so-called counterfactual thoughts are triggered by negative events where automatically alternative scenarios of the past suggest themselves (Roese, 1997; Roese, 2000; van Dijk & Zeelenberg, 2005).

One reason regret is so important for individual decision-making is because people take the possible impact of regret into account when making a decision, and it influences the evaluation of the outcome of a decision (Martinez & Zeelenberg, 2015; Zeelenberg & Pieters, 2007). For example, it is likely that a product will be less enjoyed if a negative outcome is connected to it, even though the negative outcome does not affect the product itself (Zeelenberg, 1999). In line with this reasoning, regret was also found to affect important variables in a decision task, for example by decreasing trust levels (Martinez & Zeelenberg, 2015). Other studies suggested that relational maximization is positively related to regret and relational uncertainty (Mikkelson, Hesse, & Pauley, 2016).

Zeelenberg and Pieters (2007) also propose that regret may emerge as a result of two different reasons. One reason regards the decision process itself (e.g., whether it was justifiable and wise and carefully chosen), and the other concerns the consequential outcome of that decision. Based on this, a more tangible model of regret was developed, known as decision justification theory (DJT), which states that decision-related regret consists of two components. One is the comparative evaluation of an outcome, and the other is the feeling of self-blame due to a poor choice. The two components can occur independently; for example, a person can accept the blame for a bad choice even though the outcome was good. On the other hand, it is possible to feel regret for a bad outcome even in the absence of a reason for self-blame because the decision was made carefully, was well informed, and was therefore justified (Connolly & Zeelenberg, 2002; Crawford, McConnell, Lewis, & Sherman, 2002).

Another significant part of the research on regret differentiates regret caused by action from regret caused by inaction. Although it has been discussed in depth which of the two—action or inaction—causes a stronger feeling of regret, it may be assumed that the time course of processing the feelings of regret determines which one is regretted more. Initially, actions cause more painful feelings in the short run. However, in the long term, inactions will be regretted more (Gilovich & Medvec, 1994; Roese & Summerville, 2005). A possible explanation for this is that regrets arising from inactions are numerous because of the many positive outcomes that may have transpired. In contrast to this, regret arising from actions is exhaustive, as the negative consequences are already known and are more limited (Gilovich & Medvec, 1994).

### Future misses versus past misses

Roese and Summerville (2005) conducted a meta-analysis of 11 studies in the research area of regret and concluded that life domains that cause the strongest feeling of regret are education, career, romance, parenting, the self, and leisure. These results support the Future Opportunity Principle, as they are also the areas with the greatest opportunities for change. One explanation offered for this approach is that regret typically encourages corrective action (Roese, 1997); therefore, the feeling of regret will remain as long as the opportunities for change remain open. In a situation with few opportunities to correct or change the situation, processes of cognitive dissonance reduction automatically initiate as a way to process the emotional imbalance. This in turn reduces the intensity level of regret (Roese & Summerville, 2005).

Beike, Markman, and Karadogan (2008) provide another approach to the Future Opportunity Principle: the Lost Opportunity Principle, in which they suggest that people regret mostly those opportunities where they no longer have the opportunity for change. They concluded that future opportunity enables people to imagine different ways to possibly change the outcome, which increases a feeling of hope, and this in turn reduces feelings of regret. Lost opportunities, on the other hand, make it difficult to achieve psychological closure because they can no longer change the undesired outcome.

The Dynamic Opportunity Principle offers a solution to the controversial results from these two studies (Summerville, 2011). Similar to the conflicting results in the research of action and inaction regrets, the time course will determine the focus of regret feelings. Initially, the most regret would be felt when an outcome could not be corrected; however, stronger regret feelings will eventually be dedicated to the outcomes that remain open to change. When examining the research topic itself, it is important to know whether immediate or retrospective regret is taken into consideration.

The literature in this topic provides numerous examples supporting the need for consideration of more variables when attempting to answer that question. Furthermore, many articles support the future opportunity aspect. For example, Caruso (2010) highlights that the future, with its higher controllability, gives the person the impression of being able and, most importantly, being responsible to prevent the negative outcome. Following this logical reasoning, emotional reactions to future events must be more extreme than the reactions to past events, where the responsibility to change the outcome diminishes with the inability to change the past. This would contradict the argument that future opportunities would elicit feelings of hope, as stated by Beike et al. (2008). In several experiments, Caruso (2010) provides evidence for this theory. This author shows that people feel the urge to prevent future unfairness. In his experiments, the participants were more willing to make financial sacrifices in order to prevent future unfairness than past unfairness in scenarios such as judging transgressors in a lawsuit. In Caruso’s study, (2010) the intensity levels of emotions proved to be saliently higher for future events than the emotional reactions were for past events.

Another argument raised by Wilson and Gilbert (2005) is that many decisions are based on predictions of how one will feel in the future event. Therefore, people tend to overestimate the intensity of their emotional reactions to the future consequences of the outcome because they do not consider that other events and circumstances in the future will influence their emotional condition. They also do not consider how quickly they will enter the psychological recovery process where reasons will be found for feeling less regret and negative consequences will be rationalized. Consequently, Wilson and Gilbert (2005) suggest that people tend to underestimate how soon they will initiate coping mechanisms that allow swift recovery. However, the mere fear of the subjectively overestimated emotional reaction to the future outcome indicates that people might be very motivated to make greater efforts to avoid these situations in the future.

Shani et al. (2015) provide studies with results that more strongly support the Future Opportunity Principle than the Lost Opportunity Principle, meaning that future misses are expected to have a greater impact on decision-making. As their article is the reference point for this research, we also believe that the additional variables we will observe might provide more information supporting the Future Opportunity Principle. However, these authors suggested further examination of whether the results would be the same in a separate evaluation. A separate evaluation often offers different results than a joint evaluation may, and is a way to further examine the strength of a theory or to shed further light on the factors that are important for the topic (Bazerman, Moore, Tenbrunsel, Wade-Benzoni, & Blount, 1999; González-Vallejo & Moran, 2001; Hsee & Zhang, 2004; Hsee & Zhang, 2010). As opposing results could not be completely excluded, a further examination is needed that could lead to a more complete understanding of the topic.

### Experiment overview

This experiment is a replication of a previous experiment that tested how people choose between options related to missed opportunities in the past versus missed opportunities in the future, as reported in Shani et al. (2015). The original experiment gave the participant a scenario where a choice had to be made between two importers that offer the same mug for the same price. One importer offered the mug at a discounted price in the past and the other importer will offer the mug at a discounted price in the future. As the participants had to buy the mug at that time for the regular price, it was examined from which importer a purchase of the mug would cause a stronger feeling of regret.

According to the suggestions in the further research section of their article, we modified their experiment by changing the joint evaluation into a separate evaluation. The participants had to evaluate the importers twice as they initially only received the information about one discount event, and after the presentation of a second scenario, they had to reevaluate their preferences with the knowledge of the second discount event. Shani et al. (2015) concluded that people preferred purchasing the mug that was discounted to the mug that will be discounted and that they experienced more regret purchasing the mug that would be discounted. Hence, the results clearly showed that future missed opportunities triggered more feelings of regret. Besides the separate evaluation, we additionally examined whether a future missed opportunity that is further in the future would deliver the same results and added a condition where the future discount would be offered in 6 weeks rather than in 2 weeks (Hsee & Zhang, 2010; Hsee, Zhang, Wang, & Zhang, 2013).

Accordingly, we hypothesize that

H1. In a separate evaluation, future misses will cause stronger feelings of regret than past misses.

H2. A future miss will have a stronger influence on changing people’s decisions than a past miss.

H3. Future misses with a longer time distance will cause less regret than misses in the nearer future.

For potential explanations, the participants were also asked to identify the option they would feel more responsible for with regard to missing the discount and which mug they would prefer to choose. These two questions were also retained unchanged from the original experiment in order to identify possible explanations and to increase the comparability with the original work.

## Methods

### Participants and procedure

The sample was composed of 216 students (131 females, 50.9% under 20 years old, 48.6% between 21 and 30 years old) from ten different countries, predominantly in Europe, participated in this survey that was available online. Uncompleted surveys were excluded from the analysis. They were randomly assigned (by a lottery) to one of four conditions of the 2 (order of missed opportunity: past regret presented first vs. future regret presented first) × 2 (time distance: 2 weeks vs. 6 weeks) design. In the past regret first condition, the scenario was presented as follows:

“For a while, you have been considering purchasing a coffee mug. This morning at the cafeteria you see one you like priced at 15 €. The salesperson explains that two different importers (A and B) import the mug. He further explains that because both importers wish to promote the mug, they occasionally offer it for 7.50 €. Specifically, importer A offered the mug for 7.50 € two weeks ago. Because you want the mug now, you must pay the regular price of 15 €”.

Then, participants were asked what would cause them to experience the strongest feeling of regret and what would cause them to feel more responsible for missing the discount as well as which importer’s mug they would prefer purchasing (− 1 = “Purchasing from importer A, who offered the mug two weeks ago for 7.50 €”, +1 = “Purchasing from importer B, who did not offer the mug two weeks ago for 7.50 €”, and 0 = “I would feel the same whether I paid 15 € for a mug that was sold for 7.50 € or not”).

In the second part of the questionnaire, the participants were asked to imagine that the salesperson not only tells them about importer A’s discount 2 weeks ago but also that importer B will offer the mug for 7.50 € in 2 weeks (6 weeks). They were asked to assume that they still want the mug now and must therefore pay the regular price of 15 €. Participants had to answer the same questions again while considering the new information about the second importer’s discount.

In the future regret first condition, participants first read about importer B’s discount in the future, an opportunity that they already know they will miss because they want to buy the mug now. Subsequently, they learn about importer A’s discount that they also missed but in the past. The remaining information in the scenario stayed the same.

To test for our predictions—and in line with previous research (e.g., Shani et al., 2015)—, we used descriptive statistics together with both parametric and non-parametric statistical tests. We conducted an ANOVA (between-subjects) with Games Howell post hoc tests. We also run Welch *t* tests to compare for means across conditions, as the group samples had unequal variances. Moreover, Wilcoxon tests (within-subjects) were also used to compare for the different conditions within the same scenario.

## Results

We found a significant effect of presenting only one of the two discount options on the regret scores between all four conditions, *F*(3, 212) = 16.03, *p* < .001. Post hoc comparisons appear in Table 1 and indicate that the mean scores for condition 1 (past first/2 weeks) and condition 2 (past first/6 weeks) were significantly different from condition 3 (future first/2 weeks) and condition 4 (future first/6 weeks).

**Table 1:** Regret scores for one discount between the groups

		Mean diff.	Standard error	Sig.
Past first/2 weeks	Past first/6 weeks	− .081	.121	.910
	Future first/2 weeks	− .766	.142	.000
	Future first/6 weeks	− .749	.149	.000
Past first/6 weeks	Past first/2 weeks	.081	.121	.910
	Future first/2 weeks	− .685	.149	.000
	Future first/6 weeks	− .668	.156	.000
Future first/2 weeks	Past first/2 weeks	.766	.142	.000
	Past first/6 weeks	.685	.149	.000
	Future first/6 weeks	.016	.172	1.000
Future first/6 weeks	Past first/2 weeks	.749	.149	.000
	Past first/6 weeks	.668	.156	.000
	Future first/2 weeks	− .016	.172	1.000

When participants only knew about the past discount that they had missed, they felt more regret for purchasing from this importer (*M*_past first/2 weeks_ = − 0.59, *SD* = 0.59; *M*_past first/6 weeks_ = − 0.51, *SD* = 0.70) than when they heard about a future discount that they will miss (*M*_future first/2 weeks_ = 0.18, *SD* = 0.84; *M*_future first/6 weeks_ = 0.16, SD = 0.88). Evaluating the regret scores for each discount separately, the past miss elicits more regret than the future miss does. Our prediction that future miss would be equally strong in a separate evaluation as it was in a joint evaluation could not be statistically confirmed.

**Fig. 1:**
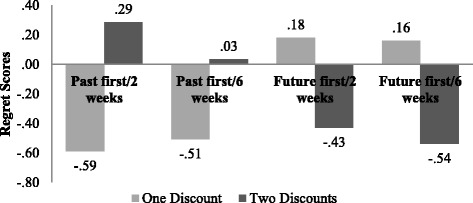
Mean regret scores for the scenarios with one discount and two discounts in all four conditions

We found a significant difference between the regret scores for the scenarios with one discount and the subsequent scenarios with two discounts within all four-condition groups. Mean regret scores for all conditions can be seen in Fig. 1. Table 2 indicates the test results and *p* values for the before and after regret scores in each group. It is clear that the mean regret scores are significantly different when introducing the second discount. Therefore, the mean difference is greater when participants first hear about the missed discount in the past and then about the future discount that they will miss (mean diff._past first/2 weeks_ = 0.88; mean diff._past first/6 weeks_ = 0.54). In comparison, the mean differences were lower when participants first knew about the missed discount in the future and then about the missed discount in the past (mean diff._future first/2 weeks_ = − 0.61; mean diff._future first/6 weeks_ = − 0.7). These results show that future miss has a stronger influence on changing people’s decisions.

**Table 2:** Wilcoxon test for before and after regret scores per condition

	Past first/2 weeks	Past first/6 weeks	Future first/2 weeks	Future first/6 weeks
*U*	− 4.912^a^	− 3.707^a^	− 3.642^b^	− 3.610^b^
Sig. (two-sided)	.000	.000	.000	.000

^a^Based on negative ranks

^b^Based on positive ranks

Merging the two past first conditions together into one and the two future first conditions into another group, we can also see that the mean difference is greater when introducing a missed future opportunity as new information. As indicated in Tables 3, 4, and 5, the difference between the before and after regret scores in the past first group is greater (mean diff. = 0.71) than in the before and after regret scores in the future first group (mean diff. = − 0.66). The additional information of a missed opportunity in the future therefore causes a stronger change in the participants’ decisions than the additional information of a past miss. It can be assumed that future miss has a stronger influence on changing people’s decisions than a past miss does.

**Table 3:** Mean regret scores by order of missed opportunity

	Mean	SD	*U*	Sig. (two-sided)
Scenario 1: missed past discount	− .55	.652	− 6.134^a^	.000
Scenario 2: missed past and future discount	.16	.894		

^a^Based on negative ranks

**Table 4:** Mean regret scores by order of missed opportunity

	Mean	SD	*U*	Sig. (two-sided)
Scenario 1: missed future discount	.17	.861	− 5.126^a^	.000
Scenario 2: missed future and past discount	− .49	.770		

^a^Based on positive ranks

**Table 5:** Regret scores for first and second scenario

Scenario 1: one discount		Mean diff.	Standard error	Sig.
Future first/2 weeks	Future first/6 weeks	.016	.172	1.000
Scenario 2: two discounts		Mean diff.	Standard error	Sig.
Past first/2 weeks	Past first/6 weeks	.252	.166	.430
Future first/2 weeks	Future first/6 weeks	.109	.154	.894

We found no significant effect of missing the future opportunity in 2 weeks’ time or in 6 weeks’ time on regret. Participants who only knew about the future discount in 2 weeks did regret purchasing from this importer slightly more (*M* = .18) than participants who knew solely about a future discount in 6 weeks (*M* = .16). However, as indicated in Table 5, this difference was not statistically significant. The prediction that future misses with a longer time distance would cause less regret than misses in a nearer future could not be statistically verified.

**Fig. 2:**
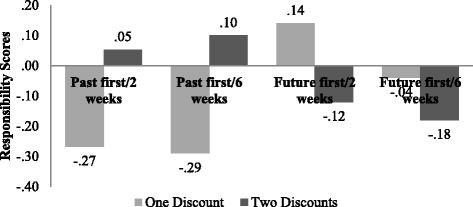
Mean responsibility scores for the scenarios with one discount and two discounts in all four conditions

We found significant differences between the two responsibility mean scores within the past first and future first conditions with a 6-week time distance. We also found a marginally significant difference within the past first condition with the 2-week time distance. Figure 2 depicts the mean responsibility scores for all four conditions. Table 6 depicts the before and after responsibility mean differences in each group and their significance levels.

**Table 6:** Wilcoxon test for before and after responsibility scores per condition

	Past first/2 weeks	Past first/6 weeks	Future first/2 weeks	Future first/6 weeks
*U*	− 1.770^a^	− 2.469^a^	− 1.528^b^	− .903^b^
Sig. (two-sided)	.077	.014	.127	.014

^a^Based on negative ranks

^b^Based on positive ranks

The overall picture of the responsibility scores is very similar to the regret scores (Figs. 1 and 2). When participants felt more regret for purchasing from the importer that offered a past discount, this same importer made them feel more responsible for missing the discount. When participants changed their mind about their regret feelings after the new information of a future discount, they were also more inclined to feel more responsible for missing the new discount. These similarities of the regret and responsibility mean score movements apply also to the conditions where the future discount was introduced first.

Between the four conditions, there was predominantly no significant difference in the mean scores. There was only a significant difference in the scenarios with one missed opportunity between the groups Past first/6 weeks and future first/2 weeks (mean diff. = − .425, SE = .161, *p* = .047, 95% CI = .00, .85). The difference of variances between the four groups for the responsibility scores is therefore assumed to be mostly within the groups. In the matter of responsibility feelings, people seem to have different perceptions or are more indifferent to feeling responsible about missing the discount from one importer or the other.

**Fig. 3:**
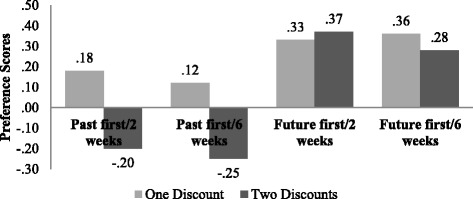
Mean preference scores for the scenarios with one discount and two discounts in all four conditions

We found a significant and marginally significant difference between the preference scores for the scenarios with one discount and the subsequent scenarios with two discounts in the past first conditions. Figure 3 depicts the mean preference scores for all four conditions. Table 7 summarizes the test results and *p* values for the before and after preference scores in each group.

**Table 7:** Wilcoxon test for before and after preference scores per condition

	Past first/2 weeks	Past first/6 weeks	Future first/2 weeks	Future first/6 weeks
*U*	− 1.915^a^	− 2.043^a^	− .243^b^	− .500^a^
Sig. (two-sided)	.056	.041	.808	.617

^a^Based on positive ranks

^b^Based on negative ranks

It can be observed that in the past first conditions, the participants changed their mind after they read the second scenario. First, they preferred to purchase from the importer that did not offer a discount in the past (*M*_past first/2 weeks_ = .18; *M*_past first/6 weeks_ = .12) and then they preferred to purchase from the importer that did offer a discount in the past rather than purchasing from the importer that is related to a future miss (*M*_past first/2 weeks_ = − .20; *M*_past first/6 weeks_ = − .25). Comparing with the regret scores (Fig. 1), the participants seemed to prefer purchasing from the importer that does not elicit as much regret.

The mean scores in the future first conditions, however, present a different picture. Participants did not change their minds when they additionally read about a past discount that they have missed. In both scenario sequences, they preferred to purchase from the importer that will offer the mug in the future at a lower price even though they felt initially more regret for purchasing from this importer. It is noteworthy that these regret scores were very close to zero (*M*_future first/2 weeks_ = .18; *M*_future first/6 weeks_ = .16), which indicates that they were more temped to be indifferent to the importer from which they more regret to purchase. This could be an explanation for the preference choices that do not show any clear decision-making to prevent a future miss or past miss.

In all four groups, the tendency was towards preferring to purchase the mug from the importer B. There were consequently no significant differences in the preference means at the first scenario round, *F*(3, 212) = .788, *p* = .502. In the past first conditions, it can be assumed that the participants chose this importer because, at that point, it was the only choice without any discount related opportunities. So they could easily distance themselves from the importer that offered a discount in the past. In the future first conditions, they preferred to purchase from the importer that offered a discount in the future. It seems that there was no strategy to distance themselves from the future miss when this is the only miss of which they are aware.

After the introduction of the second scenario, the preference mean scores differed significantly between the four conditions, *F*(3, 211) = 5.962, *p* < .01). A post hoc test shows that there were mostly significant differences between all conditions if the only difference was not the time variable. There was one marginal difference between past first/2 weeks and future first/6 weeks (mean diff. = − .480, SE = .191, *p* = .064, 95% CI = − .98, .02). It can therefore be assumed that the variance of differences is mainly between the groups. In the past first conditions, people preferred purchasing from the importer connected to a past miss, and they therefore distance themselves further from the future miss. In the future first conditions, the participants did not try to distance themselves from the future miss and preferred purchasing from the importer that offers a discount in the future.

## Discussion

Shani et al. (2015) are presumably the first authors to draw a direct comparison between regret connected to a past miss and regret related to a future miss. They showed that a possible future regret could influence people’s decision-making more than regret caused by a past miss. We tried to shed further light on these findings by testing the strength of their findings in a separate evaluation and different time variables. By presenting to the participant two sequential scenarios with only one discount in the first scenario and two discounts in the second, we were able to collect data for a separate evaluation and a joint evaluation with different orders of the missed opportunities. Thus, we could also evaluate to what extent they changed their minds when introducing a future regret as new information.

In the separate evaluation, we find that people feel more regret and responsibility for a past miss than for a future miss. This remains contrary to the findings of the study to which we are referring. Furthermore, regarding their purchase preferences, participants chose more to purchase from an importer connected to a future miss than from an importer that offered a discount in the past. This also remains in sharp contrast to the findings of the previous study. However, a future miss as second information had more influence on changing their mind about their feelings of regret than the new knowledge about a past miss did. This is informative for the strength of the Future Opportunity Principle and fulfills the prediction that a future miss is more impactful. Regarding the different periods, we found that future misses with a longer time distance did not cause less regret than misses in the nearer future did. One reason could be that the two periods were not large enough to present significant differences in the regret means. Another possible explanation is that the impact of a future miss is so strong that the feelings of regret remain stable regarding the period or are even locked to other external influences. These results have clear practical implications for both consumers and companies (as decision-making actors). Specifically, companies (e.g., retailers) should be very careful when advertising future opportunities for products or services for which justifying a future miss is difficult, as consumers may find it easier to justify missing a future opportunity for offerings that satisfy utilitarian goals than for those that satisfy hedonic goals (Shani et al., 2015).

A limitation of this research resides in the study power. Although our sample size was large enough to test for our predictions, not all of them were totally confirmed. Both parametric and non-parametric statistical analyses were conducted, in line with the previous studies of Shani et al. (2015). Future research could seek to test our predictions with a larger and more diversified sample. Specifically, future research could examine different decision-making individual profiles regarding future and past misses. For example, maximizers are known as decision makers who are as concerned with the potential loss of existing options as with the loss of undiscovered future ones (Moyano-Díaz, Martínez-Molina, & Ponce, 2014; Patalano, Weizenbaum, Lolli, & Anderson, 2015). Future research could also seek to increase the difference between the periods and further test the possibility to influence the feelings of regret with the time variable. This could be important for the corporate sector, should companies need to decide how far in advance they can announce a discount. If customers feel too much regret for missing the future discount, they will have negative associations with the product and enjoy the product less. Also, future research could seek to carry out other statistical analyses, such as moderation and mediation analyses with additional variables, as well as to adopt longitudinal randomized experiments.

The preference choices showed that participants chose to purchase mostly from the importer that will offer a discount in the future. This was also the case when they felt more regret for purchasing from this importer and the alternative was an importer that did not offer any discounts. One reason could be that the participants misunderstood the instruction and thought that they could buy the mug in the future. Shani et al. (2015) mentioned that the participants could have difficulties to imagine losing a future discount opportunity, even to the extent that they are less aware or are completely unaware of the information that they do not have the possibility to purchase the mug for the discount price. In a follow-up study, Shani et al. (2015) mentioned the participants’ persistence to accept that they cannot return the product and get the discount later, even though this was explicitly mentioned in the instructions. A method to circumvent this persistence could be to make the scenario more impossible also for the future miss, for example with a geographic restriction. The mug could be a unique souvenir from a vacation destination and the discount will be offered after the participants are flying back home. This would make it easier for the participant to accept the impossibility to fix this missed opportunity.

## Conclusions

Our results show that, when evaluated separately, future misses caused less regret than past misses. Conversely, future misses made participants change their feelings of regret more intensely than past misses did. Moreover, regret levels did not decrease when future misses were deferred. In sum, our findings support the strength of future misses on regret but also show contrasting effects when the options were evaluated separately. Thus, we did not expect a reversal of preferences or feelings of regret in a separate evaluation, yet the results stand in contrast to the results of the joint evaluation. Overall, we seek to shed some light into the comprehension of the psychological mechanisms of past and future regret in decisions that involve different time frames. Which situation elicits more regret still remains a complex issue that needs further examination. This study attempted to move one step closer to the comprehension of these phenomena.
