# Correction to: past and future regret and missed opportunities: an experimental approach on separate evaluation and different time frames

**DOI:** 10.1186/s41155-017-0077-5

**Published:** 2017-10-23

**Authors:** Luisa Papé, Luis F. Martinez

**Affiliations:** Nova School of Business and Economics, Universidade Nova de Lisboa, Campus de Campolide, 1099-032 Lisboa, Portugal

## Correction

The original publication (Past and future regret and missed opportunities, 2017) contains errors in two equations in the Result section. The original article was updated to rectify these errors.

The correct versions can be found below:

Incorrect version (p. 4 in PDF):

We found a significant effect of presenting only one of the two discount options on the regret scores between all four conditions, F(3212) = 16.03, *p* < .001.

It should read:

We found a significant effect of presenting only one of the two discount options on the regret scores between all four conditions, F(3, 212) = 16.03, *p* < .001.

Incorrect version (p. 6 in PDF):

There were consequently no significant differences in the preference means at the first scenario round, F(3212) = .788, *p* = .502.

It should read:

There were consequently no significant differences in the preference means at the first scenario round, F(3, 212) = .788, *p* = .502.
